# Periodontal Health Status Among Cigarette Smokers and Nonsmokers in Chitwan District, Nepal: A Comparative Cross‐Sectional Study

**DOI:** 10.1155/bmri/4177627

**Published:** 2026-03-10

**Authors:** Shristi Kafle, Abhishek Gupta, Erika Shrestha

**Affiliations:** ^1^ Department of Periodontology and Oral Implantology, Chitwan Medical College, Bharatpur, Chitwan, Nepal, cmc.edu.np; ^2^ Department of Oral Medicine and Radiology, Chitwan Medical College, Bharatpur, Chitwan, Nepal, cmc.edu.np

**Keywords:** cigarette smokers, community periodontal index, oral hygiene index-simplified, periodontal health status

## Abstract

**Background:**

Cigarette smoking significantly affects an individual′s periodontal health.

**Aim:**

This study was aimed at evaluating and comparing the periodontal health status between smokers and nonsmokers.

**Materials and Methods:**

A comparative cross‐sectional study was conducted with a total of 422 participants, 211 smokers and 211 nonsmokers, aged 15–74 years, selected through convenience sampling. Based on smoking intensity, smokers were also classified as light, moderate, or heavy smokers. Periodontal condition was evaluated based on the plaque index (PLI), simplified oral hygiene index (OHI‐S), and Community Periodontal Index. Data were analyzed using IBM SPSS Statistics Version 27. Descriptive and inferential statistical methods included the Chi‐square test, Mann–Whitney *U* test, Kruskal–Wallis test, and post hoc analysis.

**Results:**

The results revealed that smokers exhibited poorer periodontal conditions, marked by deeper periodontal pockets and increased clinical attachment loss compared to nonsmokers. Furthermore, the data revealed that heavy smokers experienced greater periodontal damage, followed by those who smoked moderately and lightly.

**Conclusion:**

The extent of cumulative smoking exposure and the deterioration of periodontal health were found to be significantly dose–response correlated.

## 1. Introduction

Periodontal disease, a chronic inflammatory disorder of the supporting structure of the teeth, continues to be one of the common causes of tooth loss on a global scale [1, 2]. Beyond oral health‐related consequences, periodontitis has been associated with systemic diseases, including cardiovascular events (CVDs), diabetes mellitus, and unfavorable pregnancy outcomes [1]. The burden of periodontal disease is higher in low‐ and middle‐income countries, and preventive and diagnostic measures are more frequently neglected [2].

Of the several risk factors for periodontitis, tobacco use has consistently been documented as the strongest environmental factor [3, 4]. Tobacco smoke holds several toxic components, such as nicotine, carbon monoxide, and free radicals that could perturb the oral microbial community as well as host immune responses [5, 6]. Observational studies have shown that smokers are two to eight times more likely to develop periodontal attachment loss and alveolar bone destruction when compared with nonsmokers [7, 8]. Furthermore, smoking is known to suppress classical signs of gingival inflammation, such as bleeding on probing, which complicates the early clinical detection of periodontal disease [9, 10].

A dose–response relationship between tobacco exposure and periodontal breakdown has been consistently reported across diverse populations [11, 12]. However, differences in smoking behaviors, socioeconomic status, and oral health practices emphasize the importance of generating population‐specific data [13]. In Nepal, tobacco use is a major public health issue, with high prevalence among adults and increasing uptake among adolescents and young adults [14]. Although several studies in different regions of Nepal, including Sunsari, Kathmandu, and Parsa, have documented worse periodontal outcomes among smokers compared to nonsmokers, evidence remains scarce for other districts [11–13]. Clinically, in the Chitwan District area, very limited data are available for the dose–response relationship based on smoking intensity on periodontal health that necessitated special studies to address these issues as this one [12].

Closing this lacuna in knowledge is necessary to comprehend local disease burden and direct focused preventive efforts. Establishing region‐specific evidence can be used to facilitate the incorporation of smoking cessation interventions into periodontal care in clinical and community settings.

Accordingly, the main purpose of this research was to evaluate and compare the periodontal status among cigarette smokers with that of nonsmokers in Chitwan District, Nepal. The second aim was to assess the dose–response relationship between smoking level (light, moderate, and heavy smoking) and severity category of periodontal disease.

## 2. Materials and Methods

### 2.1. Study Design and Ethical Approval

A comparative cross‐sectional, hospital‐based study was carried out over a 13‐month period, from July 2024 to August 2025, in the Department of Periodontology and Oral Implantology, Chitwan Medical College and Teaching Hospital. Participants were recruited from patients attending the outpatient dental clinic for routine dental examination or periodontal evaluation. The sample therefore represents individuals seeking dental care, rather than the general community population. Ethical clearance was obtained from the Institutional Review Committee (IRC) of the institution (Approval No. CMC‐IRC/080/081‐149).

### 2.2. Ethical Considerations and Consent Procedures

The IRC approved the use of verbal informed consent, considering the noninterventional nature of the study and the routine clinical setting. Verbal consent was obtained after explaining the study objectives and procedures in the participant′s native language and was documented in a study logbook by the examiner at the time of enrollment.

For participants aged 15–17 years, verbal assent was obtained from the participant along with verbal consent from a parent or legal guardian, as approved by the ethics committee. Confidentiality and anonymity were strictly maintained throughout the study.

### 2.3. Sample Size Determination

Sample size was calculated using the formula for comparison of two means:

n=2×σ2×Zα/2+Zβ2d2

where *σ* = 0.99 (standard deviation from prior study) [15], *Z* *α*/2 = 1.96 (for 95% confidence interval), *Z* *β* = 0.842 (for 80% study power), and *d* = 0.27 (expected effect size), representing the expected difference between group means based on prior literature.

Substitution yielded a sample size of approximately 211 per group. Thus, the study included 211 smokers and 211 nonsmokers, for a total of 422 participants. Participants were recruited according to predefined inclusion and exclusion criteria.

### 2.4. Eligibility Criteria

Eligible individuals were between 15 and 74 years of age who had at least 10 natural teeth, could understand and follow oral hygiene instructions (verbal or written), and consented/were willing to participate.

### 2.5. Exclusion Criteria

Exclusion criteria included a history of systemic disease affecting periodontal health, periodontal treatment within the previous 3 months, absence of periodontal inflammation with a community periodontal index (CPI) score of 0, or use of antibiotics, anti‐inflammatory drugs, or immunosuppressive therapy within the past 6–8 weeks. Former smokers/smokeless tobacco users/dual users (cigarette plus smokeless tobacco) were also excluded. Pregnant women and hormonal contraceptive users were also excluded.

### 2.6. Justification for Exclusion of CPI = 0

Exclusion of subjects with CPI = 0 was made to concentrate the analysis on individuals with some degree of periodontal involvement that could potentially reflect differences in disease severity between smokers and never smokers.

### 2.7. Smoking Status and Categorization

Cigarette smoking status was gathered via self‐report and participants were categorized as current cigarette smokers or nonsmokers.

• Never smoker: subjects who never smoke cigarettes
• Smokers: current cigarette smokers only
• Smokers were also divided according to the average number of cigarettes smoked per day:
  • Light smokers: ≤ 10 cigarettes/day
  • Moderate smokers: 11–20 cigarettes/day
  • Heavy smokers: > 20 cigarettes/day

This categorization was also employed to determine the dose–response relationships of smoking intensity and periodontal parameters.

### 2.8. Clinical Examination and Examiner Calibration

Proper smoking history was taken followed by a thorough periodontal examination using the mouth mirror and CPITN probe. Corroborated smoking history preceded the complete periodontal examination with a mouth mirror and CPITN probe. One examiner trained in periodontal examination performed all clinical examinations. The examiners were calibrated before examination, which means that they made an initial repeated examination of selected patients under supervision by the faculty to achieve consensus in scoring periodontal indexes. Although no official reliability statistics were obtained, calibration exercises attempted to reduce the intraexaminer variability.

### 2.9. Periodontal Parameters Assessed

Various indices like Plaque Index (PLI), Simplified Oral Hygiene Index (OHI‐S), and CPI were used to assess oral hygiene status and periodontal status and treatment needs, respectively.

In periodontal chart, debris and calculus score for Teeth #16, 11, 26, 46, 31, and 36 was recorded and OHI‐S was determined. Periodontal health status was evaluated using the CPI, as recommended by the World Health Organization. Examinations were performed with a CPITN probe and mouth mirror, beginning from the maxillary right sextant. The following CPI codes and criteria were applied [16].

• Code 0: healthy periodontium
• Code 1: bleeding observed during or after probing
• Code 2: calculus detected on probing
• Code 3: periodontal pocket 4–5 mm (black band partially visible)
• Code 4: periodontal pocket ≥ 6 mm (black band not visible)
• Code X: sextant excluded (< 2 teeth present)
• Code 9: not recorded

### 2.10. Loss of Attachment (LOA)

LOA was described as the mean CAL for each participant considering all sites per tooth and used in statistical analysis as continuous data. LOA was measured using a calibrated periodontal probe. Each tooth was divided into six probing sites as mesio‐facial, mid‐facial, disto‐facial, mesio‐lingual, mid‐lingual, and disto‐lingual. At every site, the distance from the cemento‐enamel junction (CEJ) to the base of the sulcus or periodontal pocket was measured and noted down in millimeters. The individual average LOA value from all the test sites of each subject was used as a mean LOA score for intergroup comparisons.

### 2.11. Data Management and Statistical Analysis

Collected data were coded and entered into Microsoft Excel (Version 16) and analyzed using IBM SPSS Statistics Version 27. Continuous variables were summarized as means and standard deviations. Data were screened using descriptive statistics and normality testing. Because the primary periodontal variables exhibited a nonnormal distribution, nonparametric tests were employed for all subsequent analyses.

The Mann–Whitney *U* test compared periodontal parameters between smokers and nonsmokers. Associations of smoking categories with PLI, OHI‐S, and LOA were analyzed by Kruskal–Wallis test. After the Kruskal–Wallis test, post hoc pairwise comparisons were made with Bonferroni correction for multiple comparisons. To compare proportions between groups, a Chi‐square test was used. Multivariable analyses were planned to investigate for potential confounding by factors such as age and gender. However, it was decided that unadjusted comparisons were appropriate given the exploratory nature of the study, which is acknowledged. A *p* value < 0.001 was considered highly significant, < 0.05 statistically significant, and > 0.05 not significant.

## 3. Results

A total of 422 participants were included in the study, consisting of 211 cigarette smokers and 211 nonsmokers. The age of participants ranged from 23 to 65 years. The mean age of smokers was 38.69 years (SD 9.26), while nonsmokers had a mean age of 40.38 years (SD 8.73) (Table 1).

**Table 1:** Mean and standard deviation according to age among the cigarette smoker and nonsmoker groups.

Group	*n*	Mean age	SD
Nonsmokers	211	40.38	8.73
Cigarette smokers	211	38.69	9.26

Abbreviations: *n*, number of respondents in the specific group; SD, standard deviation.

**Figure 1:**
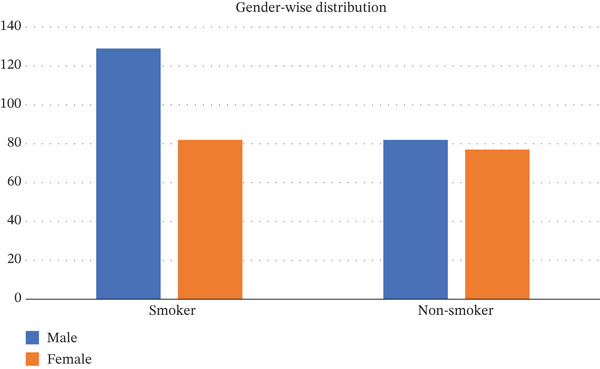
Gender‐wise distribution of study participants.

The age difference between the two groups was assessed using the Mann–Whitney *U* test and was not statistically significant (*p* = 0.08). Gender‐wise distribution is as presented in Figure 1.

### 3.1. Comparison of Periodontal Parameters by Smoking Status

Plaque accumulation was slightly lower among smokers, with a mean PLI of 1.29 (± 0.47), compared to 1.34 (± 0.38) in nonsmokers, though the difference was not statistically significant (mean difference = −0.05; *p* = 0.197). Similarly, the OHI‐S was marginally lower in smokers, that is, 2.30 (SD 0.91) than in nonsmokers, which was 2.39 (SD 0.93), but this difference also failed to reach significant level (mean difference = −0.09; *p* = 0.288) (Table 2).

**Table 2:** Comparison of periodontal parameters based on smoking status.

Parameters	*n*	Mean (SD)	Mean rank	*p* value
PLI				
Nonsmokers	211	1.34 (0.38)	219.15	0.197
Smokers	211	1.29 (0.47)	203.85	
OHI‐S				
Nonsmokers	211	2.39 (0.93)	217.91	0.288
Smokers	211	2.3 (0.91)	205.19	
LOA				
Nonsmokers	211	3.06 (1.59)	144.30	< 0.001 ^∗^
Smokers	211	5 (1.4)	278.70	

Abbreviations: LOA, loss of attachment; *n*, number of respondents in the specific group; OHI‐S, oral hygiene index‐simplified; PLI, plaque index; SD, standard deviation.

^*^Significant.

In contrast, LOA was markedly greater among smokers, that is, 5.00 (SD 1.40) compared with nonsmokers, that is, 3.06 (SD 1.59), and this difference was highly significant (mean difference = 1.94; *p* < 0.001) (Table 2). The difference in mean LOA between smokers and nonsmokers is clinically relevant and indicates greater periodontal destruction among smokers.

### 3.2. Periodontal Parameters Among Smoking Categories

Table 3 summarizes the periodontal indices across smoking categories. Light smokers demonstrated significantly lower plaque scores than moderate and heavy smokers (*p* < 0.001). OHI‐S scores did not vary significantly across smoking categories (*p* = 0.375). LOA, however, showed a progressive increase with smoking intensity: 3.32 (SD 1.16) in light smokers, 5.12 (SD 0.32) in moderate smokers, and 6.21 (SD 0.46) in heavy smokers (*p* < 0.001).

**Table 3:** Comparison of periodontal parameters among various categories of smokers.

Parameters	*n*	Mean	Std. deviation	*p* value
PLI				
HS	86	1.37	0.48	
MS	60	1.36	0.48	< 0.001 ^∗∗^
LS	65	1.12	0.42	
OHI‐S				
HS	86	2.24	0.91	
MS	60	2.30	0.97	0.375
LS	65	2.38	0.89	
LOA				
HS	86	6.21	0.46	
MS	60	5.12	0.32	< 0.001 ^∗∗^
LS	65	3.32	1.16	

Abbreviations: LOA, loss of attachment; *n*, number of respondents in the specific group; OHI‐S, oral hygiene index‐simplified; PLI, plaque index; SD, standard deviation.

^**^Highly Significant.

### 3.3. CPI Findings by Smoking Status

CPI scores revealed marked differences between smokers and nonsmokers (Table 4). A significantly higher proportion of smokers exhibited shallow and deep periodontal pockets compared to nonsmokers (Chi‐square test, *p* < 0.001). Smokers were more likely to present with CPI Code 4 (deep pockets), whereas bleeding on probing was more frequently observed among nonsmokers.

**Table 4:** CPI scores based on smoking status.

CPI scores	Nonsmoker	Smoker	Total	*p* value
	*n* (%)	*n* (%)	*n* (%)	
1	96 (22.7)	25 (5.9)	121 (28.7)	
2	93 (22.0)	67 (15.9)	160 (37.9)	< 0.001 ^∗^
3	15 (3.6)	50 (11.8)	65 (15.4)	
4	7 (1.7)	69 (16.4)	76 (18.0)	

^*^Significant.

Among nonsmokers, 22.7% exhibited bleeding on probing, 22.0% had calculus, 3.6% presented with shallow pockets, and 1.7% had deep pockets. In smokers, these proportions shifted toward more severe disease: Only 5.9% had bleeding on probing, 15.9% had calculus, and 11.8% had shallow pockets while 16.4% presented with deep pockets. When smokers were stratified according to the amount smoked (light, moderate, and heavy), a direct correlation between smoking dose and periodontal destruction was found. Mean LOA was 3.32 (37) ± 1.16 in light smokers, 5.12 (41) ± 0.32 in moderate smokers, and 6.21 (26) ± 0.46 in heavy smokers; the difference between groups was statistically significant (Kruskal–Wallis test, *p* < 0 · 001). Post hoc analysis showed that Ammon′s horn volume differed significantly between light and moderate smokers, light and heavy smokers, and moderate and heavy smokers (Bonferroni‐adjusted *p* < 0.001 for all).

### 3.4. Stratification by Smoking Intensity

Table 5 demonstrated a clear gradient as follows:

• Light smokers: 23.1% bleeding, 36.9% calculus, 21.5% shallow pockets, and 18.5% deep pockets.
• Moderate smokers: 15.0% bleeding, 33.3% calculus, 30.0% shallow pockets, and 21.7% deep pockets.
• Heavy smokers: 1.2% bleeding, 26.7% calculus, 20.9% shallow pockets, and 51.2% deep pockets.

**Table 5:** CPI scores in various categories of smokers.

CPI scores	Code 1	Code 2	Code 3	Code 4	Total	*p* value
	*n*(%)	*n*(%)	*n*(%)	*n*(%)	*n*(%)	
Heavy smokers	1 (1.2)	23 (26.7)	18 (20.9)	44 (51.2)	86	< 0.001
Moderate smokers	9 (15.0)	20 (33.3)	18 (30.0)	13 (21.7)	60	< 0.001
Light smokers	15 (23.1)	24 (36.9)	14 (21.5)	12 (18.5)	65	< 0.001

*Note:* % denotes frequency of the partcipants.

Abbreviations: CPI, community periodontal index; *n*, number of respondents in the specific group.

This pattern reflects a dose–response effect, with heavy smokers demonstrating substantially greater disease severity.

### 3.5. Post Hoc Analysis

Pairwise post hoc comparisons confirmed significant differences in both PLI and LOA between smoking categories. Light smokers had significantly lower PLI compared to moderate and heavy smokers (*p* < 0.01), while LOA increased significantly across light, moderate, and heavy smoking categories (*p* < 0.001) (Tables 6 and 7).

**Table 6:** Post hoc analysis of mean plaque index between various categories of smokers.

Dependent variable	Group comparison	Mean difference (LS‐MS/HS)	*p* value
PLI	LS‐MS	0.24 (−3.506)	0.001
PLI	LS‐MS	0.25 (3.984)	0.000
PLI	LS‐MS	0.01 (0.162)	1.000

*Note:* % denotes frequency of the participants.

Abbreviations: CPI, community periodontal index; HS, heavy smoker; LS, light smoker; MS, moderate smoker; *n*, number of respondents in the specific group; PLI, plaque index.

**Table 7:** Post hoc analysis of mean LOA between various categories of smokers.

Dependent variable	Smokers′ categories	Mean difference (LS‐MS/HS)	*p* value
LOA	LS‐MS	−1.80 (−5.944)	< 0.001
LOA	LS‐MS	−2.89 (13.456)	< 0.001
LOA	LS‐MS	−1.09 (6.822)	< 0.001

*Note:* % denotes frequency of the participants.

Abbreviations: HS, heavy smoker; LOA, loss of attachment; LS, light smoker; MS, moderate smoker; *n*, number of respondents in the specific group.

## 4. Discussion

This study evaluated the periodontal health condition of smokers and nonsmokers from Chitwan District, Nepal. Dental plaque and general oral hygiene condition of the participants, as assessed using PLI and OHI‐S, were not significantly different between the two groups. LOA, on the other hand, was significantly higher among smokers as a measure of worse periodontal destruction. The distribution of CPI also revealed that smokers had a higher prevalence of a more advanced type of periodontal disease than nonsmokers. Furthermore, upon stratification of smokers into light, moderate, and heavy smoking categories, a dose‐dependent association was evident as disease severity was associated with the increasing extent of smoking, reflected by greater attachment loss and pocket depth.

Various global as well as regional studies have reported to highlight that smokers have significantly higher prevalence and severity of periodontal diseases in relation to nonsmokers [17–19]. Patients who are smokers tend to have deeper pockets, greater clinical attachment, and bone loss, with disease severity being associated with tobacco exposure in a dose–response pattern, rather than implying causation [20–22]. This association has been reported in various populations such as those in Afghanistan, South Korea, and Europe [19, 22, 23].

Although smokers show more advanced periodontal loss, some studies state that plaque scores among smokers are not higher than those in nonsmokers or they may even have lower values [24, 25]. This paradoxical phenomenon has been mostly attributed to the masking effect of smoking on gingival inflammation [25]. For example, smokers may have similar plaque indices as nonsmokers and still experience more severe periodontal destruction [24].

On the contrary side, some previous studies have shown higher plaque and calculus levels in smokers as compared to nonsmokers which may result in their worse periodontal condition [26, 27]. These results are largely explained by behavioral disparities, such as less frequent tooth brushing, for a shorter duration and lack of vigor in oral care behaviors (smoker motivation) [27]. Socioeconomic and educational discrepancies have also been involved in the differences observed with respect to oral hygiene behavior [23].

The dose–response association between tobacco exposure and periodontal breakdown is strongly supported by previous research. Seminal studies by Bergström, Grossi, Weijden, and Alpagot, alongside recent systematic reviews and meta‐analyses, consistently show that the risk and severity increases in association with both the quantity of cigarettes smoked and the duration of smoking [18, 20, 28]. Heavy smokers have been reported to carry a five‐ to twenty‐fold higher likelihood of destructive periodontal disease compared to individuals who have never smoked [18].

Nicotine and other components of cigarette smoke exert multiple detrimental effects on the periodontal tissues through vascular, immune, microbial, and bone‐related pathways. Nicotine′s vasoconstrictive properties reduce blood flow in gingival tissues, leading to decreased gingival bleeding and masking early clinical signs of inflammation [29, 30]. Chronic tobacco exposure also induces microvascular dysfunction, characterized by structural and functional alterations in oral microcirculation, which further suppresses visible signs of periodontal disease [29].

Smoking compromises both innate and adaptive immunity, including suppression of helper T‐lymphocytes and impaired neutrophil function, thereby weakening host defenses against periodontal pathogens [30, 31]. In addition, smokers demonstrate heightened levels of proinflammatory mediators such as interleukin‐6 (IL‐6), tumor necrosis factor‐alpha (TNF‐*α*), matrix metalloproteinases (MMPs), and prostaglandin E2 (PGE2), which collectively exacerbate tissue destruction [5, 31].

The second important pathway is the subgingival microbial dysbiosis. Smoking changes the subgingival microbial ecology in that it is more favorable for pathogenic species such as *Porphyromonas gingivalis*, leading to a pathogen‐dominated biofilm which is more resistant to the host′s capabilities and treatment [32, 33]. These microbiological modifications frequently lead to clinical manifestations, indicating that dysbiosis is an early consequence of the action of tobacco [33].

At the bone level, smoking disturbs the balanced signaling between receptor activator of nuclear factor kappa‐B ligand (RANKL) and osteoprotegerin (OPG), leading to an increased production of RANKL, which then shifts the balance toward osteoclastogenesis and bone resorption [31]. Increased RANKL expression and OPG suppression in smokers lead to enhanced alveolar bone loss [31]. In addition, nicotine and carbon monoxide hamper wound healing through impairment of angiogenesis and collagen synthesis, poorer response to periodontal, and implant therapy among smokers compared with nonsmokers [5, 6].

Collectively, these biological mechanisms account for the heightened risk of periodontal breakdown, suppression of early clinical detection, and poorer treatment response in smokers when compared to nonsmokers.

Cigarette smoking is one of the most important modifiable risk factors to periodontal disease, leading to a substantial proportion of cases worldwide [22, 28]. This risk is dose‐dependent and decreases following smoking cessation, thus emphasizing the importance of early intervention strategies [28, 34].

The early diagnosis of periodontal disease is problematic in the smoking population because the classical clinical sign of gingival paresthesia (as bleeding) is inhibited by nicotine‐induced vasoconstriction. This masking effect renders it imperative for periodontal screening on smokers as a policy to find out the disease before it progresses [19, 30].

Dentists and other oral health care professionals are essential in combating smoking‐induced periodontal disease. They too are in an ideal position to screen for smoking habits, provide patients with cessation counseling, and integrate cessation‐centered approaches into everyday dental care [5, 6]. There is evidence that people who stop smoking not only lower the risk of periodontitis but also respond more positively to treatment for fungal infection [28, 35], which highlights the dual benefits of cessation when it comes to oral and overall health.

These are urgent concerns in the context of Nepal. Nepal has one of the highest prevalences of tobacco use in South Asia, and its prevalence is increasing among young people [11]. The concurrence of high prevalence of tobacco use, low public awareness about oral systemic health linkages, and lack of preventive services make focused interventions a public health imperative [14]. In Chitwan, the burden of periodontal disease might be notably diminished by integrating smoking cessation counseling into dental and primary healthcare and school‐ and community‐based interventions.

This study has several important strengths. First, this was the first comparative study to assess the periodontal status among cigarette smokers and nonsmokers in the Chitwan District, filling a critical evidence gap in the local region. Second, periodontal status was evaluated using standardized and commonly used indices (PLI, OHI‐S, and CPI). It ensured the homogeneity and reproducibility of the clinical measurements by using a sole calibrated examiner. Lastly, we had a sample size that was sufficiently powered for this study with an equal number of smokers and nonsmokers (n = 211 in each group), thereby improving the validity of comparisons without risk of selection bias.

Despite its strengths, this study has certain limitations that should be acknowledged. First, its cross‐sectional design restricts the ability to establish causal relationships between cigarette smoking and periodontal disease progression. Second, subjects were recruited by convenience sampling from a single tertiary care dental center; thus, the results may not be generalizable to individuals in the wider community. Third, the use of the CPI limited to index teeth may have led to an under‐ or overestimation of the true prevalence and severity of periodontal disease. Additionally, the study did not incorporate microbiological or radiographic assessments, which could have provided deeper insights into the microbial profile and alveolar bone status associated with smoking. In addition, exposure to passive smoking was not evaluated, leaving its potential impact on periodontal health unaccounted for. Finally, while the sample size was adequate, exact matching and multivariable adjustment for potential confounders such as age and gender were not performed, which may have introduced some residual confounding.

Based on the findings of this study, several avenues for future research are recommended. First, longitudinal cohort studies are needed to establish causal relationships between smoking and periodontal disease progression, as cross‐sectional data cannot determine temporality. Second, microbiological and immunological profiling of smokers versus nonsmokers would provide deeper insights into the biological mechanisms underlying the observed differences in periodontal status. Third, incorporating radiographic assessments could help in accurately evaluating alveolar bone loss, which is a critical marker of periodontal disease severity. Finally, studies that assess the impact of smoking cessation on periodontal healing in the Nepalese population would be valuable for guiding clinical interventions and public health strategies aimed at reducing the burden of smoking‐related oral diseases.

## 5. Conclusion

These findings are considered as baseline data on periodontal health status among smokers and nonsmokers in Chitwan, Nepal. It was found that cigarette smoking significantly correlated to the degree of periodontal disease, especially with greater LOA and advanced periodontal pocketing. Heavy smoking intensity was related to more severe periodontal involvement, showing a clear dose–response by this sample. These findings highlight the need for inclusion of regular periodontal evaluation and preventive smoking cessation counseling to the dental practice, especially among patients with higher tobacco exposure.

## Funding

No funding was received for this manuscript.

## Conflicts of Interest

The authors declare no conflicts of interest.

## Data Availability

The data that support the findings of this study are available from the corresponding author upon reasonable request.
